# “Help with rowing the boat”: Implementing and evaluating the Strengthening a Palliative Approach in Long-Term Care program in four Canadian provinces

**DOI:** 10.1177/26323524251369121

**Published:** 2025-09-07

**Authors:** Sharon Kaasalainen, Genevieve Thompson, Lynn McCleary, Lorraine Venturato, Abigail Wickson-Griffiths, Paulette V. Hunter, Tamara Sussman, Donny Li, Shane Sinclair, Thomas Hadjistavropoulos, Noori Akhtar-Danesh, Valerie Bourgeois-Guerin, Deborah Parker

**Affiliations:** 1 School of Nursing, McMaster University, Hamilton, ON, Canada; 2 College of Nursing, Max Rady Faculty of Health Sciences, University of Manitoba, Winnipeg, MB, Canada; 3 Faculty of Applied Health Sciences, Brock University, St. Catharines, ON, Canada; 4 Faculty of Nursing, University of Calgary, AB, Canada; 5 Faculty of Nursing, University of Regina, SK, Canada; 6 St. Thomas More College, University of Saskatchewan, Saskatoon, SK, Canada; 7 School of Social Work, Faculty of Arts, McGill University, Montreal, QC, Canada; 8 Faculty of Arts, University of Regina, SK, Canada; 9 Faculty of Health, University of Technology Sydney, Ultimo, NSW, Australia

**Keywords:** palliative approach, palliative care, end-of-life care, long-term care, family caregiver, dementia

## Abstract

**Background:**

Despite high mortality rates in long-term care (LTC), LTC homes continue to struggle to implement a palliative approach to care.

**Objectives:**

The objective of this research was to implement and evaluate the Strengthening a Palliative Approach in Long-Term Care (SPA-LTC; www.spaltc.ca) program. Specifically, we explored its feasibility, acceptability, and preliminary effects on resident comfort, use of emergency department at end-of-life (EOL), and location of resident death.

**Design:**

This study used an explanatory mixed method design in four LTC homes; one in each of four provinces (Ontario, Manitoba, Saskatchewan, Alberta) in Canada to assess acceptability, feasibility, and preliminary effects of the program.

**Methods:**

Quantitative and qualitative data were collected whereby the qualitative component was used to help explain or elaborate on the main quantitative components.

**Results:**

Of the 102 participating residents, 74.5% (76/102) had a Palliative Care Conference (PCC). However, of those who died, only 68.8% of them had a PCC. Rates of hospital use were reduced for study participants in terms of emergency department visits at EOL (relative risk reduction (RRR): 46%; 95% CI: −1.12, −0.10) and hospital deaths (RRR: 88%; 95% CI: −4.06, −1.12) compared to baseline. However, there were no significant differences in resident comfort. Family members stated that the PCCs were informative and thought that good communication was critical in providing quality care. They highlighted that close relationships and mutual respect among staff, residents, and families led to more meaningful care while the resident was alive as well as into bereavement. Staff stated that they found the SPA-LTC resources helpful and recognized the importance of having strong leadership using a Palliative Champion Team.

**Conclusion:**

The SPA-LTC program appears to be feasible on some key activities and supports a family-centered approach to care, which relies on strong communication. Future research is needed to confirm these initial results.

## Background

As the population ages, more people will die in long-term care (LTC) homes. In Canada, annual mortality rates of residents in LTC range from 27% to 52.3%.^1^ Similar rates have been observed internationally. In Norway, the annual mortality rate among nursing home residents was 31.8% over a 3-year period.^2^ The United States had an annual mortality rate of 35% within the first year of admission.^3^ A similar rate was also observed within Italy, where the annual mortality rate was 34% after the first year.^4,5^ Despite this, end-of-life (EOL) care is currently suboptimal in LTC with pain and other symptoms being poorly managed,^6^ in addition to large differences in prescribing rates for EOL symptom relief medications.^1^ Additionally, inattention to advance care planning (ACP)^7^ and timely EOL care planning has also been observed. Recent evidence further suggest discordance between values for EOL care and what is documented in administrative records, alongside a lack of physician engagement, and challenges in ACP teamwork/communication.^8^ Other identified challenges include inattention to loss, grief and bereavement of residents, families, staff,^9,10^ and inappropriate hospitalizations.^11–14^

In fact, hospitalizations are often unnecessary, with an estimated one in three resident emergency department (ED) visits being avoidable,^15^ and many times not aligned with resident preference. For example, a quarter of these ED visits were for potentially preventable conditions (e.g., urinary tract infections) and 10% of visits were for non-urgent reasons that did not require inpatient admission (e.g., falls).^15^

**Figure 1.**
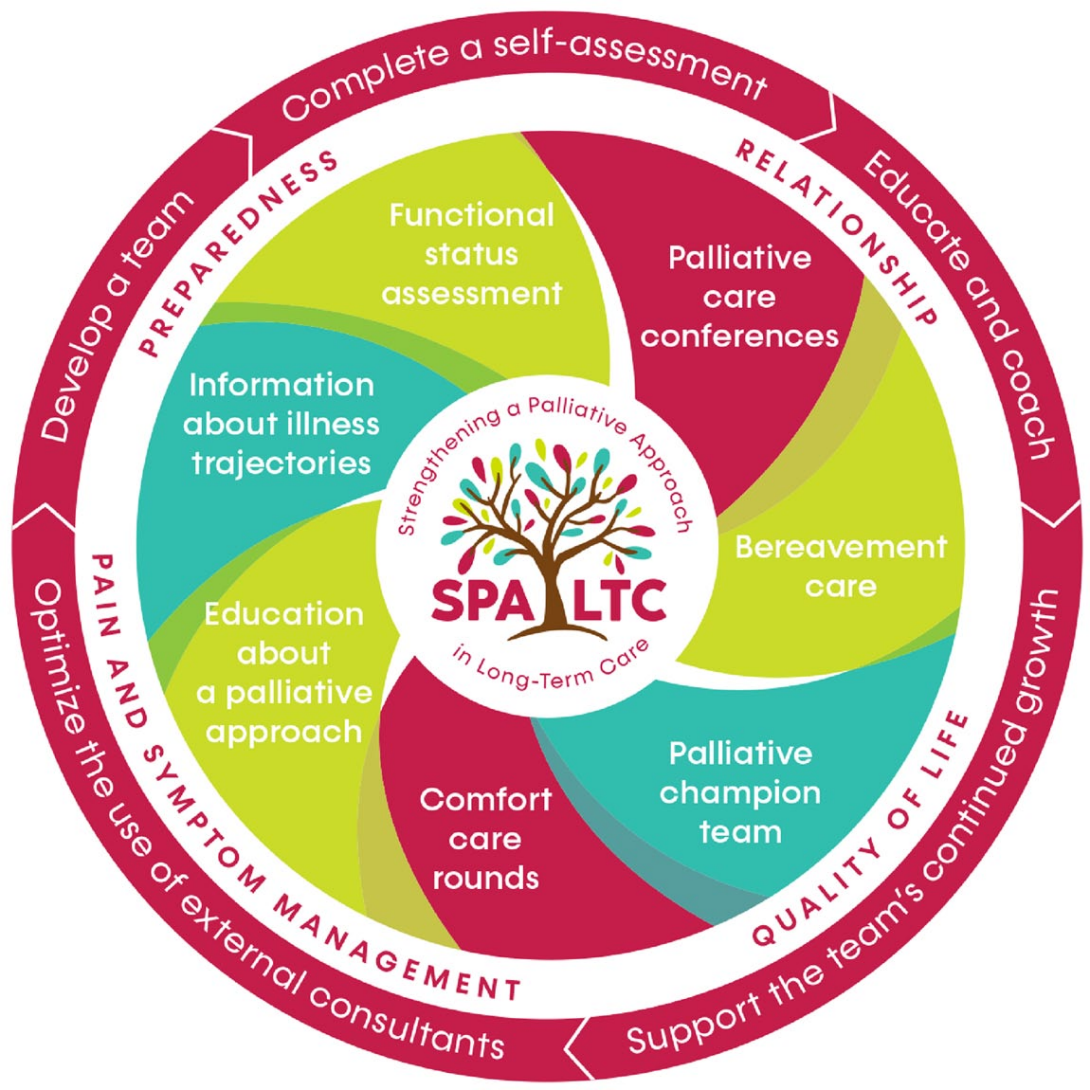
SPA-LTC program. SPA-LTC: Strengthening a Palliative Approach in Long-Term Care.

There are many barriers to optimizing a palliative approach in LTC homes. EOL care planning is complicated in LTC where most residents die from non-cancer conditions, such as co-occurring dementia, heart failure, and/or respiratory conditions,^16–19^ where prognostication is challenging. Furthermore, factors such as workload, lack of resources, staffing of LTC homes, and insufficient knowledge pose as barriers to implementing a palliative approach.^20^ Our team’s recent pilot work explored a variety of individual tools and practices aimed at improving a palliative approach in LTC,^21,22^ which led to the development of the Strengthening a Palliative Approach in Long-Term Care (SPA-LTC) program. The SPA-LTC program (see Figure 1) includes components to (a) build organizational capacity with the LTC home, by holding Palliative Champion Team (PCT) and Comfort Care Rounds (CCRs) with staff,^17,23^ (b) prepare residents and family within a palliative approach by providing an informational pamphlet soon after admission,^18^ (c) implementing the Palliative Performance Scale (PPS) as a trigger for a Palliative Care Conference (PCC),^24–27^ and (d) providing post-bereavement follow-up.^27^ The *goal of this study* was to implement and evaluate the SPA-LTC program in LTC to address the following research questions:

1. What is the feasibility and acceptability of the SPA-LTC program in four sites across Canada (primary)?
2. What is the potential effect of the SPA-LTC program in LTC on resident comfort, use of ED at EOL, and location of resident death?

## Methods

### Design

This study involved an explanatory (QUAN + qual) mixed method design based on participatory action research, whereby the qualitative component was used to help explain or elaborate on the main quantitative components.^28–30^ Mixed methods allow researchers to address complicated research questions and collect a richer and stronger array of evidence than can be accomplished by any single method alone.^29^ Furthermore, we followed the Standards for Reporting Implementation Studies (StaRI) guidelines.^31^

Since the primary goal was to evaluate the feasibility and acceptability of the SPA-LTC program when it was implemented under real-world conditions, a prospective, pre–post test design was employed with no new staff hired to implement the intervention.^32,33^ A qualitative descriptive design was used for the post-implementation component to explore perceptions about the acceptability of the intervention from the perspective of residents (if able), family members, and staff.

### Settings

We chose LTC homes with differential features so we could further understand how and under what conditions implementation and positive outcomes were supported. Given that our primary goal was to explore feasibility and not effectiveness, we chose sites that would allow for a deeper understanding of complex issues that could influence the implementation of the SPA-LTC program to help inform the design of future large randomized clinical trial. Hence, we selected four separate LTC homes across four Canadian provinces (ON, MB, SK, AB) representing the mix of characteristics (e.g., profit status, turnover, facility size) that impact the successful implementation and adoption of change efforts.^34^ See Table 1 for comparison of sites.

**Table 1.** Characteristics of case sites/LTC homes.

Characteristics	Site 1	Site 2	Site 3	Site 4
# beds	285	50	104	128
# staff	354	87	100	100
Funding model	For-profit	For-profit	For-profit	Not-for-profit
Ethno-cultural diversity	Secular, non-faith-based	Secular, non-faith-based	Secular, non-faith-based	Faith-based
Rural/Urban	Urban	Urban	Rural	Urban
Stability (i.e., administrator turnover)	2 administrators in past 4 years (moderate turnover)	2 administrators in past 2 years (high turnover)	2 administrators in past 6 years (moderate turnover)	2 administrators in past 10 years (low turnover)

LTC: long-term care.

### Participants

Inclusion criteria for residents were as follows: over the age of 65, English-speaking, and a score of 50% and less on the PPS.^22^ We chose residents over the age of 65 as they are more likely to have age-related chronic conditions and complex health needs that would benefit from a palliative approach to care. The PPS is a valid, reliable tool used to measure progressive decline in a person suffering from terminal/incurable illness, with ICCs ranging from 0.93 to 0.96 and Cohen kappa of 0.69.^35,36^ The scale can be divided into three stages: stable, 100%–70%; transition, 60%–40%; EOL, 30%; or less.^31^ We collected demographic data for all participating residents (e.g., age, gender, length of time living in LTC), Charlson Comorbidity Index (CCI; a measure that aims to categorize comorbid medical conditions that can alter mortality risk),^28^ and PPS scores.

### Implementing the SPA-LTC program

**Figure 2.**
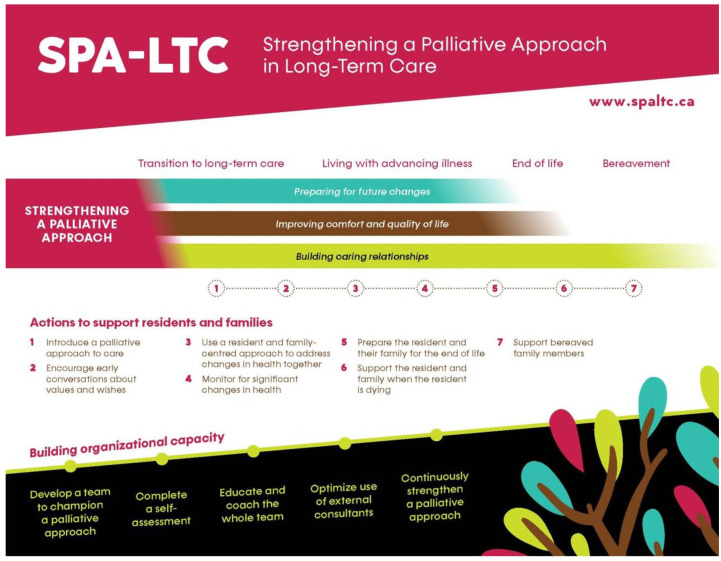
Implementation of the SPA-LTC program. SPA-LTC: Strengthening a Palliative Approach in Long-Term Care.

In this study, the SPA-LTC program builds on the team’s previous work, including components to (a) build organizational capacity within the LTC home and (b) prepare residents and family within a palliative approach. We developed a staged approach to implementing the SPA-LTC program to help offset burden and support staff with the training and skills that are needed to implement it successfully (see Figure 2).

#### Build organizational capacity

Prior to implementation of the SPA-LTC program, each site was asked to develop a PCT with representatives from all disciplines. The PCT members received training on best practices in a palliative approach, learn about the SPA-LTC program components (e.g., PPS and PCCs), and lead the implementation in study sites. Champion teams were to meet monthly to check in about implementation progress and recognize successes alongside challenges to overcome. To help maximize uptake, we encouraged sites to identify staff in leadership roles, as well as those who were passionate about palliative care and/or opinion leaders among their peers. CCRs were also available for all LTC staff on a monthly or bi-monthly basis and were intended for all LTC staff to provide a forum for case-based discussions about deceased residents or those who are dying.^17,23^ They focused on providing palliative education, reflecting on resident cases, and providing staff peer support. To help optimize staff time, they could be offered in conjunction with PCT meetings. To maximize utility, the PCT could choose the direction for the CCRs to meet the needs of staff, whether it be a forum for resident-focused discussions, palliative education, reflecting on resident deaths, or providing staff peer support.

#### Prepare residents and families

Residents who had a PPS score of 50% or less were prioritized to have a PCC. Participants (residents and families) received a printed pamphlet before having a PCC to provide information about the expected trajectory of their illness (e.g., advanced frailty, dementia, or heart, lung, or kidney disease) prompting questions from family about the availability of resources from LTC and to support ACP.^15^ PCT members also recognized the value in displaying them in visible areas for residents and families to access. PCTs were coached on best practices for holding conferences with residents and families. They were provided with PCC documents to help with (1) preparing participants for the conference (Form 1—Pre-Conference Questionnaire), (2) conference logistics (conference planning and communication form), and (3) conference content (Form 2—Conference Summary Form to prompt staff on key areas of discussion and documentation). To optimize staff, family, and resident availability, PCCs could be offered as a stand-alone conference, in conjunction with annual care conferences, or offered by telephone. After a resident participant died, families were given a bereavement informational pamphlet that addresses common reactions in bereavement and potential resources. At the champion team’s discretion, some sites provided grief and bereavement pamphlets that addressed common reactions in bereavement and potential resources through accessible palliative baskets or comfort carts, available to families when the resident was actively dying.

### Recruitment of participants in the SPA-LTC program

Research assistants worked with LTC staff to recruit residents and/or their family member who met the eligibility criteria. Once potential participants agreed to be contacted by research staff, a research assistant met with them to explain the study and obtain written consent.

### Potential effect of the SPA-LTC program

#### Hospital use at EOL and participating resident demographics

At baseline, administrative data (quantitative) were collected retrospectively for all residents who had died over the past year. Corresponding data were collected at study end, 18 months later, for participating residents only. Data were collected for the following indicators: (a) number of resident deaths; (b) number of resident deaths that occurred at the hospital versus LTC home; and (c) number of residents who visited the ED in their last year of life.

#### Comfort at EOL in dementia

Comfort was assessed at baseline and post-intervention using the Comfort at End-of-Life in Dementia (CAD-EOLD).^37^ The CAD-EOLD is a valid 14-item scale and assesses common EOL symptoms; each item is rated on a scale from 1 (not at all) to 3 (a lot). The total scale ranges from 14 to 42, with lower scores indicating better symptom control. Research supports its internal consistency (α = 0.82), convergent validity (0.50–0.81), and use in the general LTC population.^37,38^

For baseline, staff approached family members of residents who died in the year prior to the implementation of the intervention for agreement to complete the CAD-EOLD.^37^ If agreeable, a research assistant obtained consent and mailed a copy of the CAD-EOLD to bereaved family members with a return self-addressed, stamped envelope. Families were compensated with a $25 gift card for their time completing it. At post-intervention, we followed a similar process of survey completion by enrolled families whose relatives died during the intervention period.

### Feasibility of the SPA-LTC program

Feasibility of the program/intervention relates to the degree to which the participants complete and comply with the intervention.^32,33^ The feasibility was monitored using the following pre-set target criteria: (a) PPS score obtained at time of enrolment, (b) PCT meetings occurring monthly and with at least three different disciplines attending, (c) CCRs occurring monthly and with at least three different disciplines attending, (d) residents/families having a PCC before resident dies with at least three different disciplines attending, and using designated forms for documentation, (e) use of condition-specific pamphlets, and (f) use of bereavement pamphlets. We set our feasibility cutoff scores based on previous studies and accepted standards employed in implementation studies.^39^

Research assistants conducted weekly visits to complete a feasibility/fidelity checklist as well as attend monthly PCT meetings and PCCs (if permitted by resident and family). These weekly checklists were used to determine the feasibility or fidelity of each of the intervention components (see Table 4). Research assistants also completed field notes at regular intervals throughout the study to document how each site was implementing the program components, barriers, and facilitators to implementing them, so we could better understand strategies used by the homes in attempts to implement the program successfully given their local context.

### Acceptability of the SPA-LTC program

#### Family members

At study end, all family members, including both bereaved and non-bereaved of enrolled residents, were invited to participate in telephone interviews to capture their perceptions and experiences of the SPA-LTC program. Unfortunately, participating residents were unable to take part in an interview given their level of decline. Interviews invited family members to describe their perceptions of care from admission to death with a specific focus on the core elements of the SPA-LTC program (i.e., informational pamphlets, PCC, bereavement pamphlets). Questions regarding access, usefulness, adequacy of information received, and benefits of the SPA-LTC program as well as strategies for improvement in the implementation of it, were explored. For bereaved family members, we also explored their perceptions about using the bereaved pamphlets. These interview guides were developed in consultation with the study team, based on our study objectives.

#### Staff

Staff were also approached to explore their perceptions about the implementation of the SPA-LTC program and its acceptability. We worked with each site individually, to develop a strategy to arrange the focus groups in a collaborative manner. Two sites (2 and 4) organized their focus groups according to staff grouping (e.g., personal support workers/aides, registered staff, support staff), whereas the other two sites (Sites 1 and 3) combined all staff into the same focus groups. Support staff included housekeeping, kitchen, cooks, recreation, laundry, and dietician aides. For all four sites, we sought out members of the PCT to gather their feedback since they were most involved with the implementation process. Specifically, we asked staff about their perceptions of implementing the two family-directed program components (i.e., informational pamphlet, attending a PCC) along with staff-directed program components (i.e., PCT, holding CCRs). We also probed their general impressions about implementing the program, barriers that they experienced and suggestions to make the program more user-friendly to implement it in LTC. Interview guides were developed in a similar manner as the family ones, with a particular focus on implementation. We conducted 12 focus groups in total; three at each site with LTC staff including personal support workers/health care aides, registered staff/administrative staff, support staff that included the PCT members from each participating site.

#### Analysis

All interviews, field notes, and meeting notes were managed with Dedoose, a secured web-based qualitative software program for multiple users in different locations. Content analysis of all of these data involved careful reading of it to develop an initial coding framework.^40^ Important concepts that emerged from the data were labeled, categorized, and coded. Initial coding of each focus group was done independently by two individuals to foster credibility and dependability. Any discrepancies were reviewed by the investigators and discussed until consensus was reached.^41^

For the quantitative data, means and standard deviations (continuous), frequencies and percentages (categorical) were reported. Relative risk reduction (RRR) and confidence intervals (CIs) were calculated for the proportion of resident deaths that occurred in hospital as well as for ED use comparing the baseline (all residents) and post-implementation (participating residents only) data. For the bereaved family survey (CAD-EOLD), an independent *t* test was calculated to compare the difference from baseline to post-intervention.

We used the qualitative findings to help explain the study outcomes (e.g., feasibility indicators of the SPA-LTC program, hospital use at EOL, resident comfort at EOL). We integrated both the quantitative and qualitative findings to help build explanations using narratives to describe “why” and “how” these processes and outcomes occurred the way they did for each individual site/LTC home.^28^

## Results

### Characteristics of residents

Of the 102 residents who participated in the SPA-LTC program, the average age was 88.2 (SD = 7.5) years. The majority were female (77.2%) and widowed (48%). Most participants were Christian (66.7%). Participants lived in LTC for an average of 2.71 (SD = 2.9) years.

The average CCI score was 5.6 (SD = 1.5) with most (58.8%) having a PPS score of 30 or 40% (see Table 2).

**Table 2.** Characteristics of participating residents (*N* = 102).

Resident demographics	Total		Site 1		Site 2		Site 3		Site 4	
	*N* = 102		*n* = 32		*n* = 11		*n* = 21		*n* = 38	
	*n* (%)	*M* (SD)	*n* (%)	*M* (SD)	*n* (%)	*M* (SD)	n (%)	*M* (SD)	*n* (%)	*M* (SD)
Age										
In years		88.2 (7.5)		86.1 (5.8)		87.9 (7.8)		90.0 (10.2)		89.1 (6.7)
Sex										
Female	78 (77.2)		21 (65.6)		8 (72.7)		16 (80.0)		33 (86.8)	
Male	23 (22.8)		11 (34.4)		3 (27.3)		4 (20.0)		5 (13.2)	
Marital status										
Widowed	48 (47.1)		6 (18.8)		7 (63.6)		15 (71.4)		20 (52.6)	
Married	27 (26.5)		8 (25.0)		3 (27.3)		2 (9.5)		14 (36.8)	
Single	6 (5.9)		0 (0.0)		1 (9.1)		2 (9.5)		3 (7.9)	
Divorced	3 (2.9)		1 (3.1)		0 (0.0)		1 (4.8)		1 (2.6)	
Undocumented	18 (17.7)		17 (53.1)		0 (0.0)		1 (4.8)		0 (0.0)	
Religion										
Christian	68 (66.7)		6 (18.8)		11 (100.0)		13 (61.9)		38 (100.0)	
Buddhist	5 (4.9)		0 (0.0)		0 (0.0)		5 (23.8)		0 (0.0)	
Other	0 (0.0)		0 (0.0)		0 (0.0)		0 (0.0)		0 (0.0)	
No religious affiliation	3 (2.9)		0 (0.0)		0 (0.0)		3 (14.3)		0 (0.0)	
Not documented	26 (25.5)		26 (81.3)		0 (0.0)		0 (0.0)		0 (0.0)	
Ethnicity										
Europe	27 (26.5)		11 (34.4)		9 (81.8)		0 (0.0)		7 (18.4)	
Asia	2 (2.0)		1 (3.1)		0 (0.0)		0 (0.0)		1 (2.6)	
Indigenous to North America	1 (1.0)		0 (0.0)		1 (9.1)		0 (0.0)		0 (0.0)	
Latin, Central, or South America	1 (1.0)		0 (0.0)		0 (0.0)		0 (0.0)		1 (2.6)	
Undocumented	71 (69.6)		20 (62.5)		1 (9.1)		21 (100.0)		29 (76.3)	
Length of Stay in LTC										
In years		2.7 (2.9)		1.8 (1.3)		2.4 (2.0)		3.3 (2.8)	3.2 (3.9)	
CCI										
		5.6 (1.5)		5.8 (1.5)		5.0 (0.8)		5.9 (2.2)		5.5 (1.2)
PPS score*										
<30%	4 (3.9)		0		1 (9.1)		2 (9.5)		1 (2.6)	
30%–40%	60 (58.8)		18 (56.2)		6 (54.5)		7 (33.3)		29 (76.3)	
50%–60%	30 (29.4)		10 (31.3)		4 (36.4)		8 (38.1)		8 (21.1)	

CCI: Charlson Comorbidity Index; LTC: long-term care; PPS: Palliative Performance Scale.

* Percentages (%) do not add up to 100 due to missing data.

### Potential effect of the SPA-LTC program

Baseline chart audits revealed that 26.6% (151/567) of all LTC residents living within the four participating homes died in the year prior to study implementation, with the majority of the 151 resident deaths (71.5%) occurring at the LTC home (Table 3). Of the 151 residents who died, 50.3% of them visited EDs during the last year of life. Site 3 had the greatest improvement in both an absolute reduction in hospital deaths (−36.4%) and ED visits at EOL (−47.7%), while Site 2 and 4 had little improvements in ED visits at EOL (Site 2: +7.1%; Site 4: −1.1%).

**Table 3.** Differences in ED visits in the last year of life and location of death.

Variable	All sites (*N* = 567)			Site 1 (*n* = 285)			Site 2 (50)			Site 3 (*n* = 104)			Site 4 (*n* = 128)		
	Before (*n* = 151)	After (*n* = 44)	AR	Before (*n* = 58)	After (*n* = 12)	AR	Before (*n* = 14)	After (*n* = 4)	AR	Before (*n* = 33)	After (*n* = 8)	AR	Before (*n* = 47)	After (*n* = 20)	AR
Deaths (%)	26.6	43.6	16.9	20.4	38.7	18.4	28.0	36.4	8.4	31.7	38.1	6.4	35.9	52.6	16.7
In hospital (%)	18.5	2.3	−16.3^a^	15.5	8.3	−7.2	14.3	0	−14.3	36.4	0	−36.4	10.9	0	−10.9
At LTC home (%)	71.5	97.7	16.3	84.5	91.7	7.2	85.7	100	14.3	63.4	100	36.4	89.1	100	10.9
ED visits in the last year of life (%)	50.3	27.3	−23.1^b^	58.6	25.0	−33.6	42.9	50.0	7.1	72.7	25.0	−47.7	26.1	25.0	−1.1

AR: absolute reduction; RRR: relative risk reduction; ED: emergency department; LTC: long-term care.

a RRR = 88%, 95% CI: −4.06, −1.12.

b RRR = 46%, 95% CI: −1.12, −0.10.

At study end, the same data were collected for participating residents only. Of them, 74.5% (76/102) had a PCC during our 18-month data collection period, with 68.2% (30/44) having a PCC before they died (Table 4). Findings indicate that there were statistically significant reductions in rates of hospital deaths (RRR: 88%, 95% CI: −4.06, −1.12) and ED visits (RRR: 46%, 95% CI: −1.12, −0.10) during the last year of life for residents who participated in the SPA-LTC program. There were no statistical differences in resident comfort using the CAD-EOLD between baseline to post-intervention (*t* = 0.81; *p* = 0.42; Table 5).

**Table 4.** Feasibility and fidelity indicators assessed over 36-month intervention period.

Indicator	Study result				ALL	Pre-set criteria for success of feasibility
	Site 1	Site 2	Site 3	Site 4		
Palliative Performance Scale scores	88%	100%	86%	100%	93%	95% of residents have PPS scores at enrolment
Frequency of Palliative Champion Team meetings	100%	18%	64%	100%	71%	80% of homes meet monthly
Attendance at Palliative Champion Team meeting	100%	100%	100%	100%	100%	80% of homes have attendance from at least three different disciplines at each meeting
Frequency of Comfort Care Rounds	126%^a^ 21% canceled	—^b^	—^b^	264%^a^	—	80% of homes meeting monthly
Attendance at Comfort Care Rounds	100%	—^b^	—^b^	100%	100%	80% of homes have attendance from >3 different disciplines at each meeting
Occurrence of Palliative Care Conference before death	50%	50%	75%	100%	68.2%	80% of enrolled residents had a Palliative Care Conference before death
Palliative Care Conference attendance	100%	27%	100%	100%	92%	Attendance of staff from at least three different disciplines and resident/family member/friend
Palliative Care Conference form use^c^	Form 1: 88% Form 2: 100%	Form 1: NA Form 2: 100%	Form 1: NA Form 2: 100%	Form 1: NA Form 2^d^: 100%	Form 2: 100%	Forms completed for 85% of Palliative Care Conferences held
Illness informational pamphlet use	Displayed: Yes Read: 47%	Displayed: Yes Read: 47%	Displayed: No Read: 33%	Displayed: No Read: 39%		80% of homes display pamphlets onsite; 80% of residents/families received one before Palliative Care Conference
Bereavement pamphlets	16%	0%	5%	0%		80% of families who receive pamphlet

PPS: Palliative Performance Scale.

a Met more often than monthly as used huddles.

b Comfort Care Rounds were merged with Palliative Champion Team meetings.

c Form 1: Pre-Conference Family Questionnaire; Form 2: Conference Summary Form.

d Used facility-based annual care conference forms instead of Form.

**Table 5.** Change in Comfort at End-of-Life in Dementia (CAD-EOLD) from baseline to post-intervention.

Scale	Baseline, *N* = 62		Post, *N* = 27^a^		Difference	
	Mean	SD	Mean	SD	*t*	*p*
Physical distress (4–12)	7.76	2.17	7.58	1.92	0.342	0.733
Dying symptoms (4–12)	6.03	1.83	6.69	2.55	−1.140	0.260
Emotional distress (4–12)	5.91	2.26	6.37	2.09	−0.818	0.417
Wellbeing^b^ (3–9)	5.51	2.03	5.56	1.93	−0.101	0.920
Total (14–42)	23.34	6.39	24.59	5.41	−0.809	0.422

CAD-EOLD: Comfort at End-of-Life in Dementia.

a We did not break down the scores according to site due to small sample size.

b The Wellbeing subscale was reverse-coded so that lower numbers indicated a positive rating compared to those with higher scores for all subscales and total score.

### Feasibility of the SPA-LTC program

Based on the feasibility indicators that we determined before the study began, Site 4 met the most targets (8/10) whereas Site 3 met the least number of targets (2/10; see Table 4). It should be noted that the two sites who met the targets related to holding CCRs accommodated staff requests to hold more informal meetings as well, called “Huddles” that required less planning and occurred on an ad hoc basis when needed. Higher target frequencies across all sites were noted for both completing the PPS at enrolment, holding PCCs before death, and use of Form 2 during PCCs. None of the sites met two targets including: (a) use of condition-specific pamphlets; and (b) use of bereavement pamphlets.

Field notes indicated that Site 4 used their regularly occurring Multidisciplinary Annual Care Conferences as PCCs and attempted to integrate palliative content into the discussions and their regular documentation of them. In addition, it was noted that Site 2 adapted their documentation to suit telephone delivery of the PCCs, because not all families could manage the travel required to attend in-person.

### Acceptability of the SPA-LTC program

Family members reported on their perceptions of reading an informational pamphlet and attending a PCC and also provided perspective more generally about the adequacy of information and support that they received (i.e., communication and the timing of that communication). Staff also shared their thoughts about both the family-directed program components as well as the staff-directed ones (i.e., PCT, CCRs). Finally, key themes emerged from our analysis of the staff focus groups that were foundational for them to successfully implement the SPA-LTC program. These included embracing the belief that implementing a palliative approach to care needs to be interdisciplinary or that “Palliative Care Is Everybody’s Role,” and that staff need strong support from leadership and champions with their LTC home. Each of these components is discussed individually below with additional quotes included in Table S1.

### Characteristics of family and staff participants

For the acceptability component, 36 family members agreed to participate in an interview; 12 were bereaved and 20 were non-bereaved at the time of the interview. Most of them were female (84.6%), between 65 and 74 years of age (44.4%), and were a child of the resident (74.1%). The residents of these family families lived in LTC for an average of 4.6 years (SD: 5.0).

Of the 68 staff who participated in a focus group across all four sites, 96% were female, 50% were over the age of 45 years old. Most were PSWs or health care aides (51%), 17% were nurses, 15% were recreational therapists, and 15% were housekeepers or cleaners. The average length of time they had been working in LTC was 10.8 years (SD = 9.3).

### Perceptions about the informational pamphlets

#### Informational/ACP pamphlets

##### Access to informational illness pamphlet

Interestingly, most family members did not receive an informational pamphlet, though they were accessible at all LTC homes. Thus, placing them in areas where families can see them more often may help. Those who received pamphlets were generally provided by hand (e.g., directly from staff) or by mail. One family member suggested that they be placed in a more visible area or a place in the home where families tend to be present, such as the front desk.

A staff member stated,

Most of our residents and families received one [pamphlet] in the last 2 months. Our Director of Care (DOC) went through every resident here and they identified which pamphlet would be most appropriate for them and they were sent in the mail to families. And some received them during care conferences. Site 4, Support Staff

Staff from another site used a different strategy to share pamphlets with family,

The pamphlets I think are a wonderful thing and they will stay in the hallway. . . . once we get the new version of them, I’ll just print them if I need them. I have a good number still left from before, so we’ll just use those up and then I will print the new ones, but they have been, I think they are a valuable resource for people. Site 2, Registered Staff

##### Usefulness of informational illness pamphlet

The pamphlets helped provide comfort to families by informing them of what to expect and assuring them that what they saw in their loved one was to be expected. The content and amount of information seemed to be appropriate; that is, the pamphlets contained just the right amount of information. Participants felt that the pamphlets covered the common conditions that are prevalent in LTC homes, as a non-bereaved family member from Site 3 stated, “*Yes because my mother has almost all of this, she has COPD, she has congestive heart failure she has kidney issues she has dementia, she has multiple things wrong with her.*” A registered staff member from Site 2 added, “*I think the more knowledge a person has, the less scary it is because it can be scary for people when they are seeing someone go through that [dying]*.”

##### Adequacy of informational illness pamphlet

Despite the positive feedback about the pamphlets, it was clear that families also wanted some follow-up discussion about the content and have their questions addressed. One family member from Site 2 stated:

Because I found them [the pamphlets] too frustrating in some ways. I just, again it comes back to that basic need to have conversation with someone, because not. . . . it’s kind of like having autism. It’s a spectrum. It’s all over the place. So, unless you know exactly what you are asking about, if it’s under the umbrella and you have a specific question and you know where to look, you get lost. So, I prefer having that conversation, which I have quite often with the director of care, and I get better understanding.

#### Bereavement pamphlets

Unfortunately, when family participants were probed about their perceptions of the bereavement pamphlets, none of them could recall having read one enough to provide any feedback, which is reflected in our feasibility results as 2/4 of the sites did not use them and the other two sites used them minimally. We did, however, receive some feedback from staff about using the bereavement pamphlets.

It seemed that some staff felt uncomfortable supporting bereaved family members and giving them a pamphlet. This was particularly apparent in one focus group from Site 2; a site that did not use the bereavement pamphlets: “*I just felt uncomfortable doing it. That’s all, my own personal thing*.” Registered Staff

This discomfort with supporting bereaved family members was explained by a registered staff person from Site 4 stated that “*we all come from different experiences; some with more experience with dying, others for first time.*” For Site 1, where the pamphlets were reported being used the most (16%), a staff person explained: “*We put those [bereavement pamphlets] in the palliative care basket that families get. . .I have never had anybody come and ask me about it, but, but if you are replacing them [pamphlets], clearly, they are using them*.”

### Perceptions about PCCs

#### Access

Family members felt that the PCCs provided a venue for them to learn more about their loved ones’ care and to develop a greater appreciation for members of the interdisciplinary team in attendance. It gave them some time and space to develop stronger, more caring relationships with staff that sometimes was difficult to do during normal day-to-day activities. One family member commented,

It [the PCC] had good dynamics. It was very caring. And I would say, I left that meeting with some of my angst being sort of. . . . and to this day, I still don’t know her name, but the nurse, she will always say, “Hi, how are you?” and we didn’t have that connection before. I have a special connection with all those people who were in that meeting and I think they knew my mother better too. They all care. Site 3, Non-Bereaved Family Member

A staff member describes their perceptions about the purpose of a PCC:

The goals of care are discussed during care conferences. And the doctor’s job is to explain what are the goals of care and then what are the differences for family. And if the doctors could converse that in layman’s term—that would be nice so the family can understand. But it’s always done at a care conference if, let’s say there’s significant decline and then it depends on the cognitive ability of the resident. Site 1, Staff

Having physician presence was important but unfortunately this was not the case for some, which resulted in family members not getting the information that they needed to understand clinical decisions.

Because when it came to discussing my mom’s pain levels and what we needed to do, I needed to understand the medication that she was on, why she was on the medication, what we could change and what to do to continue to make her more comfortable. . . She got to the point that we thought she was passing. And it turned out we changed some of her medication and it took about five weeks of her physical shaking uncontrollably, not being able to eat or get out of bed. And once we reversed some of the medications and changed what she was doing, she improved. So that to me was disappointing, because I’ve asked more than once that a doctor be there. I understand that they are busy, but they are the ones prescribing the medication for my mom, so I needed to know exactly what is going on. Site 3, Non-Bereaved Family Member

#### Usefulness

Overall, participants felt the PCCs were useful and provided important information to them; although some found the support and information more helpful than others (Table S1 in Supplemental Information). Unfortunately, some family members were not aware that the care conference was about EOL, leaving them unprepared and sometimes confused. As one family member describes,

I sort of left with the question, what’s this all about? What was this for? And yeah, I don’t think that’s a good thing. Especially since it’s very important and I was left just not knowing, and wondering—is this just another meeting? I was a bit indifferent to it, and I didn’t want to experience indifference at that time. Site 3, Bereaved Family Member

Family members highlighted that the PCC allowed them to discuss topics that were important to them such as pain management and comfort, use of hospital at EOL, nutrition/hydration concerns feeding issues and dietary preferences, medications, music preferences, and not wanting the resident to die alone. Another family member stated how they learned about new topics to help them make more informed decisions, for example:

One thing that stood out for me was about body secretions and the fluid and things like that and how swallowing can be difficult. And that forcing somebody to eat isn’t necessarily a good thing, you know? And I found that very interesting and very helpful. Site 3, Non-Bereaved Family Member

Family members felt that the PCC was critical in helping families prepare for the EOL,

Preparing us for it . . . to prepare me and the family about the decisions that I had to make at that meeting to what kind of care that she would have. And that was explained pretty good. It prepared me. . . . and you have to start thinking that way even though you don’t want to think that way. But you have to start thinking of these decisions. And having explained about what would happen and how they would do things and all the things available to us and everything, that really did help. It was comforting to know that there were choices still. Site 3, Non-Bereaved Family Member

Staff described how a PCC provides the space and time to share information work more closely together in a caring and compassionate manner with open communication, describing how during a PCC, “*you appreciate the context that the family shared with all of us because we were just in this room for a very short, brief moment of time with these families, and what they shared was so profound and very important. . .they’re people, and I mean we treat disease so well but we also have to treat people just as well right? And it was good honest communication, compassionate communication*”—Site 3 Registered Staff.

Staff from Site 1 described how the documentation forms helped them get a better sense of what information was important for them to learn about and how it helped with communication among staff:

And you know, and certainly for me, it likely helped more than anybody else because for me it helped with that documentation. Right? To document that this person was at this space in their life or this level of care.

#### Adequacy of information and support

Two main themes emerged related to the adequacy of information and support for family members: (a) effective communication about the purpose of the meeting, specifically around “The Phone Call” [when staff call family to arrange a PCC due to recent significant decline of a resident], and (b) the timing of care conferences or discussions, namely that the timing of the PCC needed to take into consideration family receptivity to the information.

##### Communication

Some participants felt that the way in which the PCC was initially communicated to family members was important to consider, in order to avoid misunderstanding and unnecessary distress. In particular, there seemed to be some worry over “The Phone Call” (i.e., communicating that the resident is dying) and at times, family members felt they were inadequately informed about the purpose of the conference, leaving them feeling unprepared. For example, one family member stated,

I got a call about the conference and EOL. It kind of scared me, because they said please call us we need to talk about your mother’s end-of-life. And I thought something had happened. So, then I clarified and they said no, no. . . . I really actually didn’t know the specifics of it until I got there. Because I had just seen my mom and she was fine. And I didn’t connect it to what I had done several years ago or the conversations I had with the previous person. So, I thought my mother was in distress. Site 3, Bereaved Family

##### Timing

The notion of time was very apparent throughout the interviews in different ways. Participants highlighted the importance of not feeling rushed and the need to have time for more discussion.

I think it should take place when people aren’t in the midst of losing their loved one. It’s too stressful at that time when you are not focusing. So, for things to be put in place and how things are done is definitely a huge plus, because for some people they might not want to go there. But you have to go there. And for some people, this might be the first time that they thought about the funeral or what the person is going to look like or how they are going to. . . you know? And that’s enough, I think, for some people it might almost be part of the grieving, that you go home and you have a good cry about it. And I did think about it afterwards. And this is going to happen. Site 3, Non-Bereaved Family Member

Many participants spoke about the need to have care conferences more often and on an ongoing basis to help prepare them better instead of just having one toward the EOL.

I don’t know if whether a 2-step might be better. So, whether they do a refresher . . .because maybe a second time around, you would have some questions. . .because taking in all the information was hard. But it’s something that you have to know, it was information that you needed to hear. So, I was happy with the information. It alleviates your concerns to know how they deal with the person when they know the end is really near. . . . I don’t know whether evaluating my mother first and then having it might have been better. The doctors said she’s got 2 weeks to 2 months. And then you’re just dealing day-to-day with what’s going on. Site 3, Non-Bereaved Family Member

Another family member supported the need for more than one meeting as well given how difficult and complex these discussions can be:

Because these things are sensitive and there is so much to cover, it’s a first meeting and everything, I think sometimes the simplicity of it even though it’s not simple, it’s still better less than more until you are more equipped to deal with it, because definitely if you had a second meeting, it would be where I would want specifics, because I know more now and there are certain things that I would have brought up maybe with diet and stuff like that at a second meeting. I don’t think I would have done that at a first. . .smaller is better for me. Site 3, Non-Bereaved Family Member

Staff spoke of informal communication when they could not arrange a formal PCC in time:

I also find that lately there’s been a lot of little too late, last minute calling the family and it should have been sooner. Site 4, Support Staff

### Staff perspectives about other SPA-LTC components

Generally, staff gave the impression that they were quite positive about implementing the SPA-LTC program with some program components being more well-received than others. A participant from the Site 3 stated, “because I can honestly say that looking back at the years where we didn’t have something like this [palliative program] set in place, we were lacking in palliative care towards the residents and towards EOL. You gave us really good tools and resources to expand to help the comfort and the needs of the residents and the families.”

Staff highlighted how important it was to spend time in the beginning stages to prepare and support staff for the implementation process which contributed to the success of implementing a palliative approach to care.

Because I’ll be honest, before we really got ramped up into this, absolutely nobody in the facility like the front-line staff, I can confidently say did not want to have a discussion with the family regarding Palliative Care at EOL. There was not that confidence or skill base there unless they were forced into the situation, into matters of circumstance. So that’s kind of where I felt that went good. . . your consistency coming back into the home for meetings. . .to me had a huge impact because it was. . .you provided guidance. . .we needed someone to *help row-the-boat for us*. Site 3, Staff

However, staff felt that more direction was needed in terms of “what to do when”:

I think the program in and of itself is doable in all long-term care homes if it’s modified. It needs to be a written program that says, you do this PPS on admission. You do it under these conditions again. . .this should drive your discussions with families. You should know what it does, what it is, how it helps. . .because staff manage best when things are written as an actual procedure. Right? So, you know I made things up on the fly and did things as my own procedure because no procedures existed. Site 2, Staff

#### The Importance of the PCT

All of the sites had struck up a PCT early on in the implementation process and felt that it was an important strategy to implement a palliative program successfully, since it allowed staff to “assess where they’re coming from and how they would be willing to grow, evolve and take ownership and commitment to that team [PCT]. . .the philosophy behind the champion team is definitely the proper way to run it”—Site 3, Staff.

Participants described different processes for recruiting members or joining a PCT themselves; some were appointed by management while others volunteered based on their interests and relationships with others who were part of the PCT. Most often, PCT were an esteemed group of peers who were seen as leaders at the home, as echoed during a Site 3 focus group:

Well I chose myself to sit on it and I looked around the people who I thought would have the compassion to sit on this team and I had a little bit of input when it said, who. . .and then a list of names was here and I gave him a list and said, these are the people who in my opinion have the compassion and the empathy and sympathy to deal with families and residents. . .the professionalism of the people sitting on the team. Like I can walk up to any of these people sitting in this room right now and know that they can tell if I need a hug and I can tell if they need a hug. And the talk that we do amongst each other, that’s what makes it easy. The people that I work with make it easy.

Registered staff in Site 2 compared their PCT to the wound care team by leveraging the expertise of the LTC homes’ champions to support other staff who are providing the hands-on care to residents.

I do wound dressings all the time. I do the measurements and stuff like that, but I always consult [expert in home] and talk to her about wound changes, what I’m seeing and what I should be doing for it. If I have concerns, I bring her in. To me, that’s the same thing we could do with this [PCT], if we have our champion team, we can consult with them as the process goes on but ultimately the eyes and ears come down to the people who are seeing it every day. I see those dressings every day.

A staff person from Site 4 described some of the activities that they engaged in during a focus group:

I’m on the committee [PCT] and we meet I’d say maybe 3 times a year. You just go through the list of everyone on the floors and see who is palliative and if we think if anyone will be palliative in the next 3 months. So, I say: I think she’s declining or going downhill and then they’ll look into that. Then they would probably call a huddle or whatever. But it’s pretty much whoever in the next 3 months.

#### CCRs or huddles

Some staff preferred having huddles instead of CCRs as they found them easier to integrate into their everyday practice. One PSW in Site 4 stated: “*We take more from those end-of-life meetings [CCRS] where we just get together for 15 minutes. We talk about specific residents or a specific situation while we still have a chance to do something to make them more comfortable*.”

Huddles also provided a forum to discuss PPS scores of residents on a routine basis, with the subsequent documentation ensured greater follow-up. “*The RN goes over the [PPS] scoring and then goes through each department to makes sure that each is doing their part. We do it as a group in those huddles*”—Site 4, Registered Staff.

## Discussion

This study has revealed that implementing the SPA-LTC program was acceptable in four provinces across Canada, its feasibility varied across sites, and it helped minimize hospital use at EOL for residents. It also adds to our understanding of the processes and structures that promote successful implementation of a palliative program in LTC homes. Finally, it uncovers some critical features that are needed to create a sustainable change through strong interdisciplinary approach and leadership support to “Help row the boat.”

These study findings provide preliminary support for the effectiveness of the SPA-LTC program in reducing hospital use at EOL for residents, which is promising. These findings are similar to a previous pilot evaluation of the SPA-LTC program.^27^

It is noteworthy to find that having strong communication and therapeutic relationships among residents, families, and staff can buffer other challenging issues when they emerge and leaves families feeling more positive and supported despite some unfortunate negative outcomes, such as resident pain. In a systematic review, Gonella et al. found that family described good EOL care that includes keeping the family informed, promoting family understanding, and establishing a partnership with family by involving them in shared decision-making.^42^

Communication is important beyond EOL and into bereavement as well. However, it was clear from both the feasibility and acceptability findings that staff were not comfortable with supporting families during bereavement, as evidenced in part by the low rates of using the bereavement pamphlets. The qualitative findings help explain why the rates of using bereavement pamphlets were so low. Future work is needed to help build capacity among staff to engage more fully in supporting both family and residents into bereavement.

We did not find any improvement in resident comfort for participating residents. Perhaps, the CAD-EOLD is not the most appropriate outcome measure to assess the effectiveness of a program such as SPA-LTC. Moreover, the reliance on proxy report (i.e., family/friend) may have contributed to its low response rate (50%) since it needs to be completed after a resident dies, during the bereavement period. The acceptability of using this measure in LTC needs to be explored further.

Van den Block et al. found similar results in a large European cluster randomized clinical trial that used the CAD-EOLD as the primary outcome to assess the effectiveness of implementing the PACE (Palliative Care for Older People) Steps to Success program.^43^ Given the SPA-LTC and PACE programs have a strong emphasis on supporting families in their decision-making, perhaps a more family-centered tool may be more beneficial and sensitive to the changes anticipated with implementing the SPA-LTC program. Or perhaps using a survey about patient experience or to measure compassion in the LTC home would yield more meaningful findings.

It was clear in our qualitative findings that preferences related to the timing of ACP and goals of care discussions should be individualized based on the comfort and readiness of families to engage in these discussions. Providing residents and families with information in a gentle way early on to encourage contemplation and then allowing them to initiate conversations with staff when they are ready appears to work well given the findings of this study.^44^ Participants in this study also suggest that once a family member or resident received a pamphlet, then scheduling a follow-up meeting with them might be of benefit, so families can discuss the content with staff and have any of their questions addressed in a timely manner. It was clear though, that having ongoing and regular “check-ins” or discussions is needed. Also, it seems like families would benefit if PCCs were explained more to them and in a more sensitive manner to avoid the negative reactions to “the phone call.”

Tools like the Conversation Starter Kit seem to be effective in promoting residents asking questions to staff about their health and future care.^45^ However, eventually families need to make decisions, when the resident is unable, so holding PCCs is critical to allowing families the space and time to raise concerns and engage in shared decision-making with staff, including the physician. PCCs are one of the key components of the SPA-LTC program and have been shown to reduce hospital use in this study as well as a previous one.^27^ Other investigations that were conducted in the United Kingdom and internationally showed that families reported a significant reduction in family carer uncertainty in decisional conflict about care of residents after attending a PCC.^46,47^ Together, these studies highlight that holding PCCs has a significant impact on families, and thus, should become standard practice in LTC homes.

The feasibility of implementing most of the SPA-LTC components was not supported although one site (Site 4) met most of its targets. It is interesting to note that this site used their regularly occurring Multidisciplinary Annual Care Conferences and attempted to integrate palliative content into the discussions and their regular documentation of them. Moreover, this site worked with the LTC staff and adapted the CCRs into “Huddles” to help offset the demands of holding an additional meeting, which likely led to the successful implementation of the SPA-LTC program. Finding ways to integrate the ACP and bereavement pamphlets into everyday practice may improve access to them as well, either placing them in a display board on a common hallway or handing them out to families during admission or care conferences.

These study findings highlight the need to have an embedded palliative program in LTC as opposed to a stand-alone program. As such, important discussions that include ACP can be used as stepping stones so residents and families are not unprepared for “the phone call,” but rather, it becomes one of many conversations along the way. As an embedded palliative program, discussions with families about common topics like food and nutrition, music preferences that occur during everyday events, lend themselves to more comfortable and natural conversations in a supportive environment. However, complex interventions, like embedding a palliative program, create additional barriers to implementation, as realized in similar studies (e.g., PACE).^48^

Finally, our findings highlight how having strong leadership support and champions within the LTC home are critical to both implementing change and sustaining change in practice related to implementing a palliative approach to care. Such change needs to be institutional priority, where regular coaching and mentorship of staff is provided, which could include experts outside of the LTC homes if needed. Oosterveld-Vlug et al. found similar implementation and maintenance issues where their PACE coordinators played a key role in the success of the program but lack of policy, time, and staff within the LTC home impeded their efforts.^48^ Moreover, they found that the hierarchy between professional groups posed additional challenges in some countries.

### Limitations of the study

This study has limitations, including a small sample size and absence of a control group limit the interpretation and generalizability of our findings. As well, the diversity within our sample was limited, and documentation of ethnicity was not found in most of the charts. We were not able to assess the feasibility for recruitment as we were dependent on LTC staff to track the number of potential participants contacted, those that responded and their response, and it proved too burdensome for staff. Hence, we focused our feasibility assessment primarily on implementing the SPA-LTC program. Future work should include a more rigorous evaluation within the context of a controlled design and recruitment of a more diverse sample.

## Conclusion

The SPA-LTC program appears to be acceptable and supports a family-centered approach to care, which relies on strong communication. The reduction in hospital use at EOL for residents shows preliminary support for the effectiveness of the SPA-LTC program. The need for an embedded palliative program into everyday practice for staff is paramount to create a more supportive pathway for families and residents beginning from the moment they move into LTC to their final days and into bereavement. In this manner, staff need to be well supported by leadership in LTC to feel like they have the help they need to “Row the Boat.”

## Supplemental Material

sj-docx-1-pcr-10.1177_26323524251369121 – Supplemental material for “Help with rowing the boat”: Implementing and evaluating the Strengthening a Palliative Approach in Long-Term Care program in four Canadian provinces Supplemental material, sj-docx-1-pcr-10.1177_26323524251369121 for “Help with rowing the boat”: Implementing and evaluating the Strengthening a Palliative Approach in Long-Term Care program in four Canadian provinces by Sharon Kaasalainen, Genevieve Thompson, Lynn McCleary, Lorraine Venturato, Abigail Wickson-Griffiths, Paulette V. Hunter, Tamara Sussman, Donny Li, Shane Sinclair, Thomas Hadjistavropoulos, Noori Akhtar-Danesh, Valerie Bourgeois-Guerin and Deborah Parker in Palliative Care and Social Practice

sj-docx-2-pcr-10.1177_26323524251369121 – Supplemental material for “Help with rowing the boat”: Implementing and evaluating the Strengthening a Palliative Approach in Long-Term Care program in four Canadian provinces Supplemental material, sj-docx-2-pcr-10.1177_26323524251369121 for “Help with rowing the boat”: Implementing and evaluating the Strengthening a Palliative Approach in Long-Term Care program in four Canadian provinces by Sharon Kaasalainen, Genevieve Thompson, Lynn McCleary, Lorraine Venturato, Abigail Wickson-Griffiths, Paulette V. Hunter, Tamara Sussman, Donny Li, Shane Sinclair, Thomas Hadjistavropoulos, Noori Akhtar-Danesh, Valerie Bourgeois-Guerin and Deborah Parker in Palliative Care and Social Practice
