# Supplementary material for: “Help with rowing the boat”: Implementing and evaluating the Strengthening a Palliative Approach in Long-Term Care program in four Canadian provinces
Source: Palliat Care Soc Pract. 2025 Sep 7;19:26323524251369121. doi: 10.1177/26323524251369121 (PMC12415342; doi:10.1177/26323524251369121)
Supplement: sj-docx-2-pcr-10.1177_26323524251369121 – Supplemental material for “Help with rowing the boat”: Implementing and evaluating the Strengthening a Palliative Approach in Long-Term Care program in four Canadian provinces [file sj-docx-2-pcr-10.1177_26323524251369121.docx]

**Table S1: Themes Related to the Acceptability of the SPA-LTC Program with Supporting Quotes**

| **SPA-LTC Component** | **Theme** | **Supporting Quote** |
| --- | --- | --- |
| Informational Pamphlets | Access | I never noticed them, that doesn’t mean that they…I mean by all means they were there if they said they were there.…like if they were to be displayed anywhere, they should have been displayed at the front desk right when you walk in… you don’t go to the activity room when you have a parent and they’re dying.   - Site 2, Bereaved Family Interview 1   I’ve always found that people are quite reserved. They take their information. and may come back months later with a question that you don’t even know is related to this. Right?   - Site 2, Focus Group   No, they gave it to me… I would go in and I would stop at the desk of the Head Nurse and have a chat   - Site 2, Bereaved Family Interview |
|  | Usefulness | Just to know that what we were experiencing was expected and normal, if you can say normal in that circumstance.   - Site 4, Bereaved Family Interview   I had never dealt with anybody with Dementia before. It did point out, I was going to say long term, like what to expect in the future type of thing.   - Site 2, Bereaved Family Interview   It was very good because it just gave me assurances of things like it’s hard for her to speak or make eye contact, or recognition. So, it just sort of eases the worry that this is the stage of progression.   - Site 1, Non-bereaved Family Member Interview |
|  | Adequacy of Information | I thought it was precise, it was no more than one needed to know, I think.   - Site 3, Bereaved Family Member Interview   Very good pamphlet, I have to say. The pamphlet itself was very helpful. And this is what was presented to me at the meeting. … it was very helpful with some things that we would have never thought of or understood that way. So absolutely that was. It was laid out really nice too. They didn’t kill you with 50 paragraphs for every heading. I mean, you get tired when you are reading it. So this is fairly light reading and easy to understand.   - Site 3, Bereaved Family Member Interview   Frailty is not something that I had ever heard of before. But when I read what it was, it was like, oh that makes sense, because it’s almost like a combination of things. It’s not just one thing which is what Ivy has. She has a number of different illnesses. So it made sense.   - Site 4, Non-bereaved Family Member Interview |
| Palliative Care Conferences | Access | I really appreciated that they didn’t exclude any member of their team. Like for instance in their Care Conference that they have, everybody was there   - Site 4, Bereaved Family Interview   I was really well aware of what was going on which was good…just to keep the families informed of what’s going on…I’m hoping that all people are treated the same and it doesn’t matter how big or small a facility is.   - Site 2, Bereaved Family Member |
|  | Usefulness | Oh, it was excellent. It was an eye-opener. Like hey. I wasn’t expecting the help that they gave to us. This is what is going to happen. And it was enlightening. It was nice to know that somebody was going to look after her and look after the family. It was great.   - Site 3, Bereaved Family Interview   I guess alright. Like I mean I didn’t expect them to do anything. They just gave me some information. Most of which I already knew.   - Site 4, Bereaved Family Interview   It was kind of like a show. We got you in here and this is what we are doing and this is the plan and this is where we are going to move forward. But when you walk out the door, some things might happen and a whole lot of them didn’t. You know what I mean? So it was just like, don’t put me through this and then not finish it through to the end.   - Site 3, Non-bereaved Family Member Interview   And it’s always about the communication, and that is what we are always very concerned about and to make sure that everybody is knowing everything, including us of what is going on and nothing is happening without us knowing what’s going on. So that was what was my focus going in there with knowing that now OK, I’m going to be having to make more decisions for mom when it goes to the…when it develops to…. when she gets to that point.   - Site 3, Non-bereaved Family Interview   “Yeah it was definitely interesting sitting in there [at the PCC], learning all these things about the residents and then even seeing the connections the families had with them and meeting the families. Because sometimes you know the resident and it’s so much different when you meet the families. Then you understand them better.”   - Site 3, General Staff Focus Group |
|  | Adequacy of Information and Support | The staff was upfront and we were comfortable and directing the care based on what we talked about and what we knew dad would want.   - Site 4, Bereaved Family Interview   **Preparing families through better communication**  It was the communication with the staff I felt was very encouraging to ask to know what to expect.   - Site 4, Bereaved Family Member Interview   Because I didn’t realize it was an end of life conference, I guess my needs were not addressed because I was not informed.   - Site 3, Bereaved Family Interview   At that time we didn’t really understand what it [the conference] was all about.   - Site 3, Bereaved Family Interview   When the conference was set up, even to ask me to come in previous to the conference and say, we are going to have a conference and this is what it is going to be about, because your mom is coming close to this. So we want you to be comfortable coming into this meeting. We want you to be aware of what this meeting is going to be, and we want to give you time to have any questions prepared so it’s not a bomb. That would have helped a great deal. Instead of me just fluttering around   - Site 3, Non-Bereaved Family Member Interview   **Timing**  To me it was too early. I wouldn’t mind having it again…there was lots of information but I was sort of bombarded and I wasn’t in a proper state of mind then… it sort of blindsided me.   - Site 3, Non-Bereaved Family Member Interview   For my mom it was almost too late. It was…it’s something, when I thought about it too, generally we talked about this and we don’t speak so openly about death in our culture. And I’m really seeing now, and…I don’t know if you remember, but I lost my husband too. But how important it is to have these conversations before these illnesses come. Because it almost makes it harder, because contending with that as well, especially if it’s something like dementia, which is scary enough. And then to have the death conversations which is scary, because sometimes my mother, I will say a few things and she will say, I don’t want to talk about it. That kind of thing. Sometimes I’ll talk about who has already transitioned and it’s all about love and that kind of thing. Trying to comfort and give that. But it is opening up more in our world, but I really see a need for it well before we ever get to this point.   - Site 4, Non-Bereaved Family Member Interview |
